# Challenging stigma and promoting mental health literacy in junior professional sports: an evaluation of an informational event

**DOI:** 10.3389/fspor.2026.1807417

**Published:** 2026-04-09

**Authors:** Johanna Kaiser, Julie Pieper, Annalena Fesser, Fabian Felix Münch, Julian Schmitz

**Affiliations:** 1 Institute of Psychology, Faculty of Life Sciences, Leipzig University, Leipzig, Germany; 2 Institute of Psychology, Faculty of Social and Behavioral Sciences, Jena University, Jena, Germany

**Keywords:** help seeking, mental health literacy, mental health stigma, prevention, young elite athletes

## Abstract

**Background:**

Young elite athletes face unique developmental, academic, and performance-related demands, which can increase their vulnerability to mental health difficulties. Despite these risks, stigma and limited knowledge about mental health often inhibit help-seeking. Evidence-based, preventive interventions tailored to this population remain scarce.

**Methods:**

The present study evaluated a 90-minute informational event designed to enhance mental health literacy and reduce mental health stigma among young elite athletes. A total of 81 participants aged 12–18 completed self-report questionnaires before and after the intervention. Outcomes assessed included stigma-related knowledge, direct and indirect stigmatizing attitudes, psychological services literacy, and general and source-specific help-seeking intentions.

**Results:**

The findings indicate that a brief, low-threshold informational event was associated with higher levels of young elite athletes’ knowledge of available psychological support services, operationalized as psychological services literacy (*p* < .001), as well as increases in general help-seeking intention (*p* = .001) and source-specific help-seeking intention (*p* < .001). No significant changes were observed in direct (*p* = .390) or indirect stigmatizing attitudes (*p* = .179). Overall, participants reported high satisfaction with the content, structure, and delivery of the event.

**Discussion:**

The findings indicate that a brief, low-threshold informational event was associated with higher levels of young elite athletes’ knowledge of available psychological support services and their willingness to seek help. While changing stigmatizing attitudes may require more intensive or repeated interventions, the study highlights the potential of targeted preventive approaches to promote psychological well-being in junior professional sports.

## 1 Introduction

The daily lives of young elite athletes are characterized by intense training schedules, high academic demands, limited opportunities for recovery, and constant performance expectations (1, 2). Chronic pressure and sustained stress increase the risk of developing mental health problems (3). Despite this, mental health remains insufficiently addressed within professional sports contexts (4). Societal and sporting cultures often portray athletes as mentally and physically resilient, and young elite athletes may internalize this ideal from an early age, avoiding the expression of psychological distress or “weakness” (5).

Contrary to the common assumption that elite athletes are largely protected from mental health problems, recent research indicates that conditions such as depression and anxiety disorders are as prevalent as in the general population (6). Certain disorders, including eating disorders and attention-deficit/hyperactivity disorder (ADHD), appear to occur at even higher rates (7–10). However, most studies have focused on adult elite athletes, leaving younger populations comparatively understudied (11).

Emerging evidence suggests that adolescent elite athletes may be particularly vulnerable. They experience higher levels of chronic stress than non-athletic peers, especially regarding work overload, social isolation, and persistent anxiety (12). While participation in sports can support young people's physical, social, and personal development (13, 14), the structural and performance-related demands inherent in competitive sports may act as additional stressors that negatively affect mental health (15).

Adolescence is a sensitive developmental period, during which approximately 50% of all mental disorders in the general population first manifest (16). Young elite athletes face multiple stressors during this phase, including high training loads, insufficient recovery, and prolonged psychological strain, which can contribute to overtraining, injuries, and long-term health consequences (2). In addition, performance pressure, high expectations from coaches and caregivers, and highly structured social hierarchies may increase vulnerability, including the risk of abuse or maltreatment (5, 17).

These cumulative stressors can have serious consequences, such as impaired performance, reduced enjoyment of sport, premature dropout, and the development of mental health disorders (18–20). Providing young elite athletes with accessible and supportive resources is therefore essential to promote well-being. Addressing these challenges requires understanding the barriers that prevent athletes from recognizing mental health problems and seeking support.

Two central barriers to help-seeking in professional sports are stigma and low levels of mental health literacy (21). These factors are closely interrelated, as limited knowledge about mental health can reinforce stigmatizing beliefs and attitudes (22). Stigma refers to negative beliefs, attitudes, and stereotypes directed toward individuals with mental health problems, and it comprises cognitive, affective, and behavioral dimensions, including stigma-related knowledge, attitudes, and discriminatory behaviors (23). Public stigma may be internalized, resulting in self-stigmatization, whereby individuals adopt societal stereotypes and apply them to themselves (24). In professional sports, this process may lead athletes to perceive mental health problems as incompatible with their identity as resilient performers (25).

Stigma has significant negative consequences, including reduced self-esteem, impaired psychological well-being, and a diminished willingness to seek professional help (26). Stigmatized individuals often report shame, hopelessness, and social isolation, alongside reduced motivation and concentration resulting from untreated mental health concerns (27). In the athletic context, these effects may impair performance, highlighting the need to address mental health stigma in professional sports.

Low mental health literacy constitutes a second major barrier. Insufficient awareness of early warning signs and symptoms may prevent athletes from recognizing their own need for support and knowing where to seek help (21). Conversely, higher levels of mental health knowledge and familiarity with treatment options have been shown to positively influence help-seeking intentions (28, 29). Mental health literacy also encompasses awareness of support structures and the normalization of help-seeking in professional sports (21). Role models can play a key role in challenging the notion that seeking help reflects weakness, and strong social support networks are associated with increased help-seeking intentions (21, 30). In this study, we operationalized the knowledge component of mental health literacy specifically as psychological services literacy.

Taken together, these factors highlight the importance of interventions that target both mental health literacy and stigma to effectively promote help-seeking and psychological well-being among young elite athletes. Various strategies have been proposed, including education-based and contact-based approaches, which have demonstrated effectiveness in enhancing knowledge, attitudes, and help-seeking behaviors (31–34). However, the existing evidence mainly concerns athletes aged 18 and older, leaving younger athletes relatively overlooked.

To date, few mental health interventions have been developed specifically for young elite athletes, and empirical evaluations remain scarce. This lack of age-appropriate interventions is concerning, as stigmatizing beliefs often emerge in early childhood and consolidate during adolescence (35). Research on mental health in competitive sports has largely focused on adults (6), highlighting the need for targeted investigations in younger athletes.

The present study evaluates an informational event designed to increase mental health literacy and decrease mental health stigma among young elite athletes. Specifically, the intervention aims to provide knowledge about the development of stress and mental health problems, increase awareness of early warning signs, inform athletes about available support services, and promote a positive help-seeking culture.

Based on these aims, the current study examines whether participation in the event affects key indicators of the two targeted constructs:

1. Mental health literacy, reflected in knowledge about available support services and help-seeking pathways, and
2. Mental health stigma, reflected in stigma-related knowledge and direct and indirect stigmatizing attitudes.

It is hypothesized that participation in the event will lead to:

- H1: increased psychological services literacy;
- H2a: improved stigma-related knowledge;
- H2b: reduced direct stigmatizing attitudes;
- H2c: reduced indirect stigmatizing attitudes;
- H3a: increased general help-seeking intentions;
- H3b: increased source-specific help-seeking intentions.

Finally, participant satisfaction with the informational event and potential moderators of change (e.g., prior mental health exposure) were examined exploratorily to gain insight into the intervention's effectiveness and identify areas for refinement.

## 2 Materials and methods

### 2.1 Participants

Participants were recruited through German sports schools, various sports clubs, and a local support network for mental health in junior professional sports. Inclusion criteria required participants to be aged 10–18 years, provide written parental or legal guardian consent, and have sufficient German language proficiency to complete the questionnaires. Participation was voluntary, and all participants were informed about their rights and the study procedures. Ethical approval was obtained from the local ethics committee in March 2023.

Although the predefined age range was 10–18 years, no registrations were received from 10- and 11-year-old athletes. Consequently, the final sample comprised participants aged 12–18 years (*M* = 14.94, *SD* = 1.49), with a slight female majority (56.8%). In total, 145 young elite athletes completed the baseline assessment (T1), and 118 completed the post-intervention assessment (T2). Of these, *N* = 81 young elite athletes completed both the baseline (T1) and post-intervention assessments (T2). They represented 15 different sports disciplines, with the most common being judo (*n* = 21), track and field (*n* = 16), and soccer (*n* = 11).

### 2.2 Procedure

The informational event was developed, implemented, and evaluated within the framework of LIFENET, a university-based initiative of the Psychotherapeutic University Outpatient Clinic for Children and Adolescents at the University of Leipzig. The initiative aims to promote, maintain, and restore the mental health of young elite athletes. The event was designed to increase mental health literacy and reduce mental health stigma, based on established destigmatization approaches (36, 37) and tailored to the specific needs and challenges of junior professional sports.

A pre-post intervention design was employed, with assessments conducted at baseline (T1) and post-intervention (T2). Outcome measures included stigma-related knowledge, direct and indirect stigmatizing attitudes, psychological services literacy, and general as well as source-specific help-seeking intentions.

The intervention consisted of a structured slide presentation combined with interactive elements, such as quizzes, to engage participants and promote learning. Educational and contact-based components were integrated, including four pre-recorded video messages from prominent elite athletes, who shared their personal experiences with sport psychology and psychotherapy. These videos were shown during the session and did not allow for live interaction with participants, making the contact indirect. This component was intended to provide relatable examples and normalize help-seeking, complementing the educational and interactive elements of the event.

Content addressed sport-specific stressors in junior professional sports and highlighted the relationship between mental and physical health. Key messages emphasized that mental stress can negatively affect athletic performance, that mental illness is not a personal failing, and that seeking help early is both important and a sign of strength. Participants were informed about common early warning signs and available support services, empowering them to take proactive steps to maintain their mental health and well-being.

Parents or legal guardians were informed about the study procedures, aims, and participation requirements via letters distributed through schools, clubs, and local networks. Data collection occurred between June 2023 and July 2024 using the online survey platform Unipark. Participants completed an informed consent procedure for pseudonymized data, followed by a unique code to link responses across time points. The survey, which took approximately 5–10 min, included self-report questionnaires assessing study outcomes.

Participants completed the baseline questionnaire (T1) prior to the informational event, including demographic and sport-related variables and prior mental health exposure. Thirteen informational events were conducted on-site at cooperating schools and clubs, with group sizes ranging from 8 to 40 participants. Each session lasted approximately 90 min and was delivered by project management or trained student staff. After the event, participants received a handout summarizing key information and completed the post-intervention questionnaire (T2), which included the same items as the baseline assessment as well as satisfaction ratings. When internet access was unavailable, participants were provided with a QR code for later completion.

The time interval between the baseline (T1) and post-intervention (T2) assessments varied due to logistical differences in data collection. The median interval was 1.39 h (IQR = 1.29–4.55 h), with intervals ranging from 1.12 h to 5.06 days.

To ensure ongoing support, participants received contact information for several services, including a specialized consultation service for young elite athletes (LIFENET), a crisis chat via WhatsApp, and a German helpline, providing additional mental health resources.

### 2.3 Psychometric measures

#### 2.3.1 Prior mental health exposure

Participants’ prior experience with and knowledge of mental illness were assessed using two self-constructed items. One item asked about prior experience with mental illness and/or psychotherapy, and the other assessed knowledge of mental health conditions. Items were rated on a 5-point scale from 1 (“very low”) to 5 (“very high”), with higher scores indicating greater prior experience and knowledge.

#### 2.3.2 Stigma-related knowledge

Stigma-related knowledge was assessed using the Mental Health Knowledge Schedule [MAKS, (38)]. The MAKS comprises two segments: the first six items evaluate mental health knowledge relevant to help-seeking, recognition, support, employment, treatment, and recovery. A sample item is: “People with mental illness can recover.” The second segment assesses recognition of specific disorders, such as bipolar disorder and schizophrenia. Items 7–12 were excluded, as these conditions were not covered in the informational event. Items were rated on a 5-point Likert scale (1 = “strongly disagree” to 5 = “strongly agree”), with “don’t know” recoded as 3. Higher scores indicate greater stigma-related knowledge. In the original study, the first six items showed moderate internal consistency (Cronbach's *α* = .65). In the present sample, internal consistency was very low (*α* = −.07 at T1 and .35 at T2*;* McDonald's *ω* = .27 at T1 and .46 at T2), likely reflecting conceptual and age-related challenges.

#### 2.3.3 Direct stigmatizing attitudes

Measured using a subscale of the Mental Health Literacy Scale [MHLS, (39)]. One item (“People with mental illness are dangerous”) was excluded. The remaining eight items were rated on a 5-point scale (1 = “strongly agree” to 5 = “strongly disagree”) and reverse-coded, so that higher scores indicate greater stigmatizing attitudes. A sample item is: “People with mental illness should be treated with the same respect as anyone else.” The MHLS has demonstrated good reliability (Cronbach's *α* = .87) and test-retest stability (*r* = .80). In the present sample, internal consistency was acceptable (*α* = .73 at T1 and .78 at T2; *ω* = .75 at T1 and .79 at T2).

#### 2.3.4 Indirect stigmatizing attitudes

Assessed with the Attribution Questionnaire for Children [AQ-8-C, (40)]. Participants rated reactions to a scenario describing a student with mental illness transferring to their class, across dimensions such as pity, perceived dangerousness, fear, blame, support for segregation, anger, willingness to help, and avoidance. Items were rated on a 9-point scale, with higher scores reflecting more negative attitudes. A sample item is: “I would feel sorry for Charlie.” In prior research, Cronbach's *α* was .70 (41). In the present study, internal consistency was high (*α* = .86 at T1 and .88 at T2; *ω* = .87 at T1 and .88 at T2).

Both MHLS and AQ-8-C were translated into German using forward- and back-translation procedures to ensure linguistic and conceptual equivalence.

#### 2.3.5 Psychological services literacy

Knowledge of psychological support services was measured using four items developed by Matzinger and Walter (42). Items were rated on a 5-point scale (1 = “strongly disagree” to 5 = “strongly agree”), with higher scores indicating greater knowledge. A sample item is: “I know where to find information about mental illness.” In the original study, Cronbach's *α* was .62. In the present sample, internal consistency was low at baseline (*α* = .55; *ω* = .56) and improved at post-assessment but remained limited (*α* = .64; *ω* = .67). The lower internal consistency at T1 suggests potential measurement error. The scale was originally developed and applied in a sample of 16–19-year-old students. However, a comprehensive psychometric validation has not been reported. Its applicability to younger adolescents (12–14 years) therefore remains uncertain.

#### 2.3.6 General help-seeking intention

Assessed with a single self-constructed item asking whether participants would seek someone to talk to in a mental health crisis. Rated on a 7-point scale (1 = “No, definitely not” to 7 = “Yes, definitely”), higher scores indicate greater willingness to seek help.

#### 2.3.7 Source-specific help-seeking intention

Assessed using an adapted version of the General Help-Seeking Questionnaire [GHSQ, (43)], asking participants whom they would approach in a psychological crisis. Nine potential sources (e.g., coaches, family) were included, with multiple responses allowed. Each source was coded dichotomously (0 = not endorsed, 1 = endorsed). A composite help-seeking index was calculated as the mean proportion of endorsed sources across the nine items (range: 0–1), with higher values indicating help-seeking from a greater number of sources. The scale was translated into German using a forward- and back-translation procedure.

#### 2.3.8 Participants’ satisfaction

Satisfaction with the informational event was measured with self-constructed items covering practical relevance, interest, novelty, and structure (5-point scale), as well as overall satisfaction and satisfaction with the speakers (7-point scale). Participants also indicated their willingness to recommend the event and provided open-ended feedback.

Full item lists are provided in the Supplementary Material.

### 2.4 Data analysis

Participant identifiers were standardized and linked across measurement points using a conservative rule-based matching procedure. Identifiers were considered a match if the Levenshtein distance was ≤1, the verification item matched, and only one possible match existed. This procedure was applied to account for minor typographical errors while minimizing the risk of incorrect linkages. Matched cases were additionally screened for temporal plausibility. Two cases with markedly prolonged T1–T2 intervals (22 days and 200 days) were excluded, as they were unlikely to capture the immediate effects of the workshop intervention.

Given that the primary focus of the study was within-person change, analyses were restricted to participants with complete data at both baseline and post-intervention (*N* = 81). No imputation procedures were applied. Additional logistic regression analyses indicated that dropout at T2 among participants who completed the baseline assessment (T1) was not significantly associated with baseline outcome measures or participant characteristics (all *p*s > .05).

Two participants at baseline exhibited implausible item-level responses on the MAKS (38) due to a technical survey issue and were excluded from the scale score computation. Due to the very low internal consistency of the stigma-related knowledge scale [MAKS, (38)] in this sample, this scale was excluded from all analyses. Consequently, Hypothesis H2a could not be evaluated in this study.

Data analyses were conducted using R [version 4.5.2, (44)] and the lme4-package (45). Linear mixed-effects models were estimated separately for each outcome measure, with assessment point (session) included as a fixed effect and random intercepts at the individual level. Random slopes were tested but not retained due to model instability and no or limited improvement in model fit. Although the intervention was delivered across 13 events, individual event membership was not recorded; therefore, event-level clustering could not be modeled.

Exploratory analyses tested gender, prior knowledge of mental illness, and prior experience with mental illness as additional fixed effects, including their interactions with session. Prior experience and prior knowledge were mean-centered. Gender was coded with female as the reference category. Each predictor was added separately to the primary model to estimate extended models (see Supplementary Tables S2–S4). Model fit (primary vs. extended) was compared using an information-theoretic approach based on Akaike's Information Criterion (AIC; see Supplementary Table S1).

Model assumptions were assessed by visual inspection of residuals versus fitted values and quantile–quantile plots of residuals and random intercepts. No substantial deviations from normality or homoscedasticity were observed (see Supplementary Figures S1–S3). Quantitative satisfaction ratings and qualitative feedback were analyzed descriptively as part of a process evaluation of the informational event.

## 3 Results

### 3.1 Main analysis

Descriptive statistics for all outcome measures are shown in Table 1. Mean scores for psychological services literacy, general, and source-specific help-seeking intention were higher at post-assessment compared to baseline, whereas mean scores for direct and indirect stigmatizing attitudes decreased between assessment points.

**Table 1:** Descriptive statistics for outcome measures.

Concept	Scale	T1		T2	
		M	SD	M	SD
Direct stigmatizing attitudes	1–5	2.24	0.650	2.19	0.725
Indirect stigmatizing attitudes	1–9	3.13	1.45	2.95	1.51
Psychological services literacy	1–5	3.42	0.741	4.01	0.607
General help-seeking intention	1–7	4.63	1.81	5.15	1.41
Source-specific help-seeking intention	0–1	0.322	0.144	0.366	0.166

Values represent mean item scores (M) with standard deviations (*S*D).

Separate linear mixed-effects models were used to examine changes in each outcome between baseline and post-assessment. Significant session effects were observed for psychological services literacy, general help-seeking intention, and source-specific help-seeking intention. In contrast, the two stigma outcomes (direct and indirect stigmatizing attitudes) did not show significant changes (Table 2).

**Table 2:** Session effects from linear mixed-effects models.

Concept	Estimate (*β*)	SE	Fixed effects		*p*
			95% CI	*t*	
Direct stigmatizing attitudes	−0.05	0.06	[−0.17, 0.07]	−0.87	.390
Indirect stigmatizing attitudes	−0.17	0.13	[−0.43, 0.08]	−1.36	.179
Psychological services literacy	0.59	0.07	[0.46, 0.72]	8.62	<.001
General help-seeking intention	0.52	0.15	[0.22, 0.82]	3.38	.001
Source-specific help-seeking intention	0.04	0.01	[0.02, 0.07]	3.87	<.001

*p*-values for fixed effects calculated using Satterthwaites approximations. Confidence Intervals have been calculated using the Wald method.

**Figure 1:**
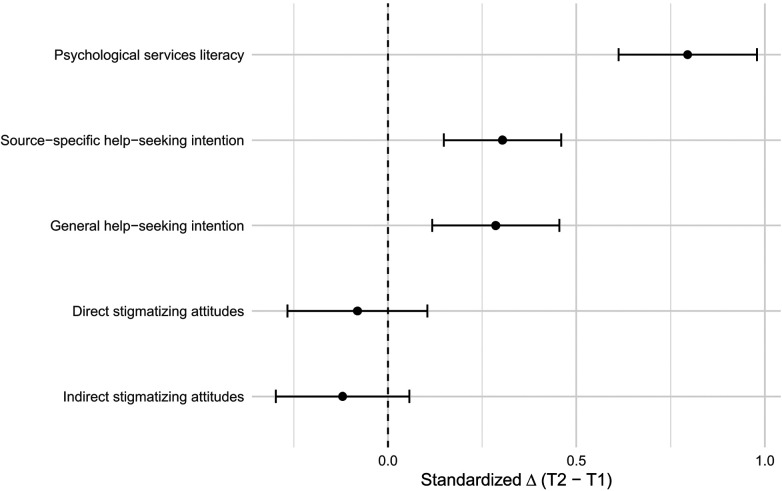
Standardized session effects across outcome measures. Points represent standardized model-based session effects (T2 − T1) with error bars indicating 95% confidence intervals. Standardization was based on the baseline standard deviation.

Figure 1 presents standardized model-based estimates of the session effect (T2 − T1) with 95% confidence intervals. Effects are reported as standardized mean differences (SMD), calculated by dividing the estimated change by the baseline standard deviation to avoid distortions due to intervention-induced changes in variability.

Psychological services literacy increased from 3.42 to 4.01 on the 5-point scale, corresponding to a large standardized effect size (SMD = 0.80).

General help-seeking intention increased from 4.63 to 5.15 on the 7-point scale, with a small standardized effect size (SMD = 0.29).

Source-specific help-seeking intention increased from 0.32 to 0.37, with a small standardized effect size (SMD = 0.30), equivalent to an average increase of approximately 0.45 sources on the nine-source measure.

Standardized effects for direct and indirect stigma outcomes were small (SMD = −0.08 and SMD = −0.12), with 95% confidence intervals overlapping zero.

### 3.2 Exploratory analysis

We examined whether intervention effects varied by participant characteristics by extending the primary model with gender, prior knowledge about mental illness, and prior experience with mental illness, each tested in separate models.

Across assessment points, several predictors showed small associations with stigma outcomes. A significant main effect of gender was observed for direct stigmatizing attitudes, with male participants reporting lower levels than female participants across assessment points (*β* = −0.31, *SE* = 0.15, *t* = −2.03, *p* = .044). Higher prior experience with mental illness was also associated with lower direct stigmatizing attitudes (*β* = −0.18, *SE* = 0.07, *t* = −2.47, *p* = .015), and higher prior mental health knowledge was associated with lower indirect stigmatizing attitudes (*β* = −0.38, *SE* = 0.19, *t* = −2.00, *p* = .048). The significance of the session effect remained unchanged in the extended models (see Supplementary Tables S2–S4). No session  ×  predictor interactions were significant (all *ps* > .353).

Adding the exploratory predictors did not improve model fit for most outcomes (ΔAIC < 2). For direct stigmatizing attitudes, including prior experience slightly improved model fit (ΔAIC = 2.91; see Supplementary Table S1).

### 3.3 Satisfaction analysis

Beyond changes in outcome measures, participants’ feedback was collected to evaluate their satisfaction with the event and identify areas for potential improvement.

**Figure 2:**
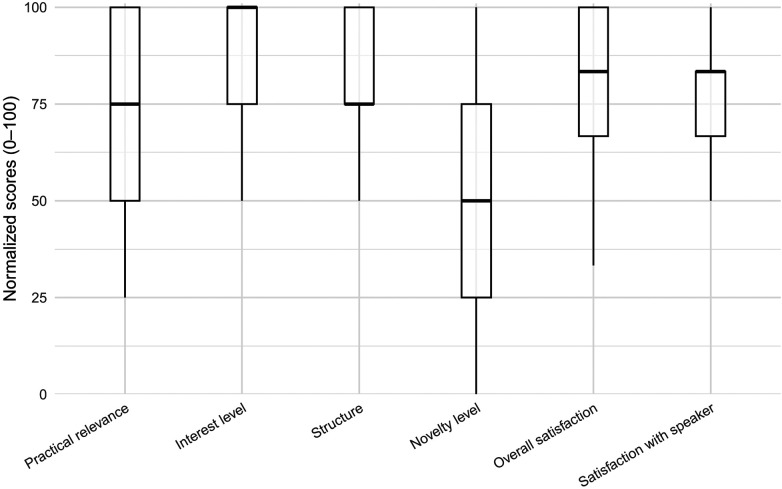
Satisfaction ratings across items. Values were rescaled to a 0–100 metric for visualization only.

The informational event received a highly positive response from participants. Ratings for the speakers (*M* = 5.78, *SD* = 0.94, scale 1–7) and the overall event (*M* = 5.86, *SD* = 1.12, scale 1–7) were high. Participants also rated the practical relevance (*M* = 3.89, *SD* = 0.88, scale 1–5), structure (*M* = 4.05, *SD* = 0.88, scale 1–5), and perceived interest of the content (*M* = 4.28, *SD* = 0.90, scale 1–5) positively. The novelty of the content received slightly lower ratings (*M* = 3.10, *SD* = 1.06, scale 1–5). Figure 2 presents boxplots of satisfaction ratings, rescaled to a 0–100 metric for visualization.

Overall, 97.5% of participants indicated that they would recommend the event to others. Open-ended feedback included constructive suggestions, such as shortening the event, providing more time for quizzes, and addressing specific questions related to personal mental health experiences.

## 4 Discussion

The present study evaluated an informational event specifically designed for young elite athletes, aimed at enhancing mental health literacy and reducing mental health stigma. Young elite athletes face unique challenges, including high training loads, academic demands, and performance pressure. These factors can increase their susceptibility to stress and mental health difficulties (2, 5). The intervention combined educational content, interactive exercises, and role model narratives to provide age-appropriate, engaging, and accessible support. Using a pre-post design, the study examined changes in knowledge, attitudes, and help-seeking intentions, contributing to the limited evidence on preventive mental health initiatives in this population.

Overall, participation in the event was associated with increases in psychological services literacy and intentions to seek help, both from general and specific sources. The large standardized effect in psychological services literacy suggests that participants left the event with substantially clearer knowledge of available support services and pathways to care. Although the standardized effect size for general help-seeking intention was small, the observed increase on the 7-point measure suggests a modest but potentially meaningful change in help-seeking intentions. Similarly, the small effect for source-specific help-seeking intention may indicate that participants considered a slightly broader range of support options following the event. These findings align with prior research suggesting that educational initiatives are associated with stronger awareness of support options and greater help-seeking behavior in youth populations (21, 46). Such programs may contribute to greater openness toward professional sources, including doctors, counseling centers, and psychologists (47, 48). By increasing knowledge about where and how to access support, the intervention may contribute to earlier recognition of mental health needs and encourage proactive engagement with appropriate services. This is particularly relevant in competitive sports environments, where stigma and self-reliance norms can inhibit help-seeking (30).

Regarding stigma-related outcomes, Hypothesis H2a, which predicted improvements in stigma-related knowledge, could not be evaluated due to very low internal consistency of the MAKS in this sample. No significant improvements were observed in direct or indirect stigmatizing attitudes, suggesting that deeply ingrained beliefs are resistant to short, education-based interventions (48, 49). This pattern is consistent with research indicating that direct contact with individuals with lived experience of mental illness is often more strongly associated with empathy, perspective-taking, and longer-term attitude change than purely informational approaches (31, 50). In the present study, the contact-based component consisted of pre-recorded videos without live interaction, representing indirect rather than direct contact. From a theoretical perspective, these observations align with models of behavior change, such as the Theory of Planned Behavior, which distinguishes between knowledge, behavioral intentions, and underlying attitudes (51). In the present study, knowledge about support services was associated with higher help-seeking intentions, while attitudes remained stable, suggesting that single-session informational events may be insufficient to influence more affectively based beliefs. These considerations are supported by evidence from multi-session educational initiatives, highlighting the potential value of repeated or more intensive contact-based components to support attitude change among young elite athletes (52).

Exploratory analyses suggested small to moderate associations between participant characteristics and stigmatizing attitudes, particularly for gender, prior experience with mental illness, and prior knowledge of mental illness. However, none of these characteristics significantly moderated intervention effects across sessions.

Feedback from participants indicated a high degree of satisfaction with the event, particularly regarding content, structure, and practical relevance. Ratings for novelty varied, providing insights for refining and optimizing the intervention. Given its feasibility and low-threshold format, the program has potential for broader application in junior professional sports. Future implementations could extend beyond athletes to include their social environment, such as coaches, supervisors, and teachers, to promote mental health awareness and support destigmatization at multiple levels.

### 4.1 Limitations and prospects

This study was conducted using a pre-post design without a control group. While this approach allowed for a more efficient and resource-saving implementation, it limits the ability to attribute observed changes solely to the intervention, as external factors cannot be ruled out. Future studies should consider including a control group to strengthen causal inferences.

A limitation of the present study concerns the assessment of stigma-related knowledge using the MAKS (38), which showed very low internal consistency in this sample (*α* = −.07 at T1 and .35 at T2; McDonald's *ω* = .27 at T1 and .46 at T2) and was therefore excluded from all analyses. This issue likely reflects conceptual and psychometric challenges in operationalizing stigma-related knowledge as a distinct construct, particularly in younger populations, as well as potential age-related comprehension difficulties. Furthermore, the relatively homogeneous level of stigma-related knowledge within this sample of young elite athletes may have contributed to the low reliability observed. Consequently, interpretations regarding the impact of the intervention on stigma-related knowledge must be made with caution.

A further limitation concerns the relatively low internal consistency of the psychological services literacy scale at T1 (*α* = .55; *ω* = .56). Lower reliability may have introduced measurement error and attenuated effect estimates. Moreover, the scale has not undergone comprehensive psychometric validation, and its suitability for younger adolescents remains unclear. Future research should employ and validate age-appropriate measures of psychological services literacy.

All outcome data were collected through self-report measures, which introduces the potential for social desirability and response biases. In addition, some of the scales employed were originally developed and validated for adult populations. Although their language and structure were judged appropriate for children and adolescents, differences in comprehension or construct interpretation may have influenced the results.

The requirement for parental and coach consent, as well as voluntary participation, may have contributed to selection bias, as athletes from more sensitized or supportive environments could be overrepresented. Additionally, participants may have had a pre-existing interest in mental health topics. This might limit the generalizability of the findings to broader or less sensitized populations of young elite athletes. Furthermore, the study was conducted within a specific national sport system, and structural as well as cultural characteristics may differ across countries and competitive contexts. Therefore, the transferability of the findings to other sport systems should be interpreted with caution.

Additionally, the variable time interval between baseline and post-intervention assessments may have introduced additional measurement noise, which should be considered when interpreting changes in mental health literacy and help-seeking intentions. Because the study did not include follow-up assessments, conclusions regarding the long-term stability of the observed effects are not possible. Future research should employ longitudinal designs with strategies to maintain participant retention. Such designs would allow for the examination of whether improvements in mental health literacy and help-seeking intentions translate into sustained behavioral changes and actual utilization of support services.

Although the workshop followed a standardized procedure, potential clustering effects cannot be ruled out, as event membership was not recorded and could therefore not be accounted for in the analysis.

The intervention combined multiple components, including didactic input, interactive elements, and video-based contact. As these elements were delivered simultaneously, their individual contributions to the observed effects cannot be disentangled. Therefore, conclusions regarding specific mechanisms (e.g., educational versus contact-based effects) should be interpreted with caution. Future research should employ component-specific or dismantling designs to clarify which elements are most effective.

Further investigation into the mechanisms underlying the observed effects is warranted. For example, disentangling the relative contributions of interactive exercises, role model narratives, and educational content could inform the development of more targeted and efficient interventions.

### 4.2 Conclusion

This study provides evidence that a brief, low-threshold informational event can be associated with increased psychological services literacy, a key component of mental health literacy, and intentions to seek help among young elite athletes. While no changes were observed in stigmatizing attitudes, the intervention was well-received and feasible to implement. These findings highlight the potential of targeted, single-session programs in junior professional sports and underscore the importance of extending future interventions to athletes’ broader social environments, including coaches, supervisors, and teachers, to promote mental health awareness and support destigmatization.

## Data Availability

The raw data supporting the conclusions of this article will be made available by the authors, without undue reservation.
