# Supplementary material for: Challenging stigma and promoting mental health literacy in junior professional sports: an evaluation of an informational event
Source: Front Sports Act Living. 2026 Apr 9;8:1807417. doi: 10.3389/fspor.2026.1807417 (PMC13102855; doi:10.3389/fspor.2026.1807417)
Supplement: Supplementary file 1 [file Datasheet1.docx]

Supplementary Material

**Supplementary Material 1**
**Questionnaire Items**

**1. Demographic Information**

**Personal ID**

In order for us to be able to match your details at this point in time with the details you provide after the information event, we ask you to create an individual ID. We cannot identify who you are based on the ID. The ID is composed as follows:

- 1st digit: Last letter of your father's first name
- 2nd digit: First letter of your mother's first name
- 3rd & 4th digits: Day of your date of birth
- 5th digit: First letter of your place of birth

Example: Father Martin, mother Kerstin, birthday April 12, born in Dresden

Code (please use capital letters): NK12D

Please enter your ID:

**What is your favorite ice cream flavor?**

*(e.g., vanilla, peach-passion fruit, cookie, double chocolate...)*

Please enter it here:

**Gender**

Male

Female

Diverse

**How old are you?**

**Which school do you go to?**

Sächsisches Landesgymnasium für Sport Leipzig

Sportoberschule Leipzig

Partnerschule des Leistungssports (Hessen)

**Where do you live during the school year?**

With my parents

At boarding school

In a shared apartment

Alone

**Are you active in competitive sports?**

Yes

No

No longer (competitive sports career already ended)

**Which sport do you play?**

**What is your current player status?**

Explanation of the answer options:

LK = State selection (federal state)

NK2 = Interface between state selection and national team, youth national team in younger age groups (15/U16)

NK1 = Youth national team in older age groups (U17-U21)

TK = National team with no prospect of qualifying for the next or second-next Olympic Games, but participation in international competitions

PK = National team with prospect of qualifying for the next or second-next Olympic Games

OK = National team with medal or final place (at Olympics, European Championships or World Championships)

**2. Prior mental health exposure**

**How would you rate your prior experience with mental illness and/or psychotherapy?**

Perhaps you know people who are mentally ill or have been in treatment yourself?

Very weak

Rather weak

Moderate

Rather strong

Very strong

**How would you rate your knowledge of mental illness?**

Please check the appropriate answer.

Very weak

Rather weak

Moderate

Rather strong

Very strong

**3. Stigma-related knowledge**

**Below are a few statements about mental illness. How much do you agree with each statement?**

Please check the appropriate box.

|  | Strongly disagree | Rather disagree | Neither agree or disagree | Rather agree | Strongly agree | I don`t know |
| --- | --- | --- | --- | --- | --- | --- |
| Most people with mental illness want to work. |  |  |  |  |  |  |
| If a friend had mental health problems, I would know what to say to encourage them to seek professional help. |  |  |  |  |  |  |
| Medication can be helpful for people with mental health problems. |  |  |  |  |  |  |
| Psychotherapy can be helpful for people with mental health problems. |  |  |  |  |  |  |
| People with severe mental health problems can make a full recovery. |  |  |  |  |  |  |
| Most people seek professional help when they have mental health problems. |  |  |  |  |  |  |

**Which of the following terms do you think represent mental illnesses?**

Please check the appropriate box.

|  | Strongly disagree | Rather disagree | Neither agree or disagree | Rather agree | Strongly agree | I don`t know |
| --- | --- | --- | --- | --- | --- | --- |
| Depression |  |  |  |  |  |  |
| Stress |  |  |  |  |  |  |
| Schizophrenia |  |  |  |  |  |  |
| Bipolar disorder |  |  |  |  |  |  |
| Alcohol addiction |  |  |  |  |  |  |
| Grief |  |  |  |  |  |  |

**4. Knowledge about support measures**

**I know where to find information about mental illness.**

Please check the appropriate answer.

Strongly disagree

Rather disagree

Neither agree nor disagree

Rather agree

Strongly agree

**I know who to turn to when I'm not feeling well mentally.**

Please check the appropriate answer.

Strongly disagree

Rather disagree

Neither agree nor disagree

Rather agree

Strongly agree

**I know what helps me in difficult times.**

Please check the appropriate answer.

Strongly disagree

Rather disagree

Neither agree nor disagree

Rather agree

Strongly agree

**It's good to address problems openly and talk about them.**

Please check the appropriate answer.

Strongly disagree

Rather disagree

Neither agree nor disagree

Rather agree

Strongly agree

**5. Attitudes toward mental illness**

**Below are a few statements about mental illness. How much do you agree with each statement?**

Check the box that best applies to you.

|  | Strongly agree | Rather agree | | Neither agree or disagree | Rather disagree | Strongly disagree |
| --- | --- | --- | --- | --- | --- | --- |
| People with mental illness can be well if they just wanted to and pulled themselves together. |  |  |  | |  |  |
| Mental illness is a sign of personal weakness. |  |  |  | |  |  |
| A mental illness is not a real medical illness. |  |  |  | |  |  |
| It is best to avoid people with mental illness to prevent developing such problems yourself. |  |  |  | |  |  |
| If I had a mental illness, I wouldn't tell anyone. |  |  |  | |  |  |
| Seeing a psychologist, psychotherapist, or psychiatrist means that you are not strong enough to cope with your own problems. |  |  |  | |  |  |
| If I had a mental illness, I would not seek help from a psychologist, psychotherapist, or psychiatrist. |  |  |  | |  |  |
| I believe that treatment for mental illness by a psychologist, psychotherapist, or psychiatrist would not be effective. |  |  |  | |  |  |

**6. Attribution Questionnaire 8-C**

**“Charlie is a new student in your class. Before Charlie's first day, your teacher explained** **that Charlie has a mental illness and is transferring from a special school.”**

Check one of the answer options for each of the following statements and questions.

**1. I would feel sorry for Charlie.**

Does not apply at all

Applies completely

**2. How dangerous would you consider Charlie to be?**

Not dangerous at all

Very dangerous

**3. How afraid would you be of Charlie?**

Not afraid at all

Very afraid

**4. I think Charlie is to blame for his mental illness.**

Does not apply at all

Applies completely

**5. I think Charlie should be in a special class for troubled teens, not a normal class like mine.**

Does not apply at all

Applies completely

**6. How angry would you be with Charlie?**

Not angry at all

Very angry

**7. How likely would you be to help Charlie with his homework?**

Extremely unlikely

Very likely

**8. I would avoid Charlie after school.**

Does not apply at all

Applies completely

**7. Seeking help**

**The following questions are about how you would respond if you were experiencing a mental health crisis.**

If you were in a mental health crisis, would you try to find someone to talk to about how you feel?

No, definitely not

2

3

4

5

6

Yes, definitely

If you were in this situation, who would you talk to?

Check all that apply.

Your best friend

Your family

Your partner

Your teacher

Your coach

Psychologist, psychiatrist, doctor

Counseling centers

Internet (people you know)

Internet (people you don't know, anonymous, e.g., forums, chat rooms)

**8. Satisfaction with the information event**

**Details about the information event**

How satisfied were you with the information event on mental health in competitive sports?

Please select the appropriate answers.

The event was relevant to me in practical terms.

Strongly agree

Rather agree

Neither agree nor disagree

Rather disagree

Strongly disagree

I found the structure of the event easy to follow.

Strongly agree

Rather agree

Neither agree nor disagree

Rather disagree

Strongly disagree

I found the content of the event interesting.

Strongly agree

Rather agree

Neither agree nor disagree

Rather disagree

Strongly disagree

The content of the event was new to me.

Strongly agree

Rather agree

Neither agree nor disagree

Rather disagree

Strongly disagree

**How satisfied were you with the speakers?**

Not at all satisfied

2

3

4

5

6

Very satisfied

**How satisfied were you overall with the information event?**

Not at all satisfied

2

3

4

5

6

Very satisfied

**Would you recommend the information event to others?**

Yes

No

**Do you have any questions, comments, or suggestions for improvement?**

We would appreciate your honest feedback.

**9. End page**

**Thank you for participating in the survey. You can now close the window.**

Further down the page, you will find contact details for support with mental health issues. Feel free to take a screenshot and get in touch if necessary.

Feel free to get in touch:

LIFENET – Johanna Kaiser

+49 341 97-35997

lifenet@uni-leipzig.de

Crisis chat via WhatsApp (24/7)

www.krisenchat.de

Nummer gegen Kummer (Mon–Sat 2–8 p.m.)

02418036777
