# Supplementary material for: Challenging stigma and promoting mental health literacy in junior professional sports: an evaluation of an informational event
Source: Front Sports Act Living. 2026 Apr 9;8:1807417. doi: 10.3389/fspor.2026.1807417 (PMC13102855; doi:10.3389/fspor.2026.1807417)
Supplement: Supplementary file 2 [file Datasheet2.docx]

Supplementary Material

**Table 1**
*Exploratory Model Comparisons across Concepts*

| Concept |  | Model comparison | |  |
| --- | --- | --- | --- | --- |
|  | Primary AIC | + Gender ΔAIC | + Prior experience ΔAIC | + Prior knowledge ΔAIC |
| Direct stigma attitudes | 293.32 | 1.54 | 2.91 | -1.42 |
| Indirect stigma attitudes | 539.77 | -1.06 | -1.12 | 1.33 |
| Psychological services literacy | 305.23 | -2.48 | -3.45 | -3.37 |
| General help-seeking intention | 580.07 | -3.88 | -3.09 | -2.96 |
| Source-specific help-seeking intention | -214.66 | -2.76 | -3.40 | -3.77 |

*Note*. Each predictor was added separately to the base model. ΔAIC values are reported relative to the primary model; positive values indicate improved model fit. Model specification for the extended models: Mean Value ~ Session × [Predictor] + (1 | ID).

**Table 2**
*Extended Linear Mixed-Effects Model Including Gender*

| Concept |  |  |  | Fixed Effects | | | |  | | |  |
| --- | --- | --- | --- | --- | --- | --- | --- | --- | --- | --- | --- |
|  |  | Term | | | Estimate (β) | SE | 95% CI | | t | p | SMD |
| Stigma direct |  | Session | | | -0.04 | 0.08 | [-0.20, 0.12] | | -0.47 | .640 | -0.06 |
|  |  | Gender | | | -0.31 | 0.15 | [-0.60, -0.01] | | -2.03 | .044 | -0.47 |
|  |  | Session x Gender | | | -0.03 | 0.12 | [-0.27, 0.21] | | -0.27 | .787 | -0.05 |
| Stigma indirect |  | Session | | | -0.23 | 0.17 | [-0.57, 0.11] | | -1.35 | .182 | -0.16 |
|  |  | Gender | | | -0.56 | 0.33 | [-1.21, 0.09] | | -1.70 | .093 | -0.39 |
|  |  | Session x Gender | | | 0.13 | 0.26 | [-0.38, 0.64] | | 0.50 | .617 | 0.09 |
| Psychological services literacy |  | Session | | | 0.55 | 0.09 | [0.38, 0.73] | | 6.09 | < .001 | 0.75 |
|  |  | Gender | | | -0.19 | 0.15 | [-0.48, 0.11] | | -1.22 | .224 | -0.25 |
|  |  | Session x Gender | | | 0.08 | 0.14 | [-0.19, 0.35] | | 0.59 | .559 | 0.11 |
| General help-seeking intention |  | Session | | | 0.57 | 0.20 | [0.16, 0.97] | | 2.76 | .007 | 0.31 |
|  |  | Gender | | | 0.05 | 0.37 | [-0.67, 0.77] | | 0.13 | .895 | 0.03 |
|  |  | Session x Gender | | | -0.11 | 0.31 | [-0.72, 0.50] | | -0.35 | .729 | -0.06 |
| Source-specific help-seeking intention |  | Session | | | 0.05 | 0.02 | [0.02, 0.08] | | 3.53 | < .001 | 0.37 |
|  |  | Gender | | | -0.01 | 0.04 | [-0.08, 0.06] | | -0.25 | .806 | -0.06 |
|  |  | Session x Gender | | | -0.02 | 0.02 | [-0.07, 0.02] | | -0.93 | .353 | -0.15 |

*Note.* *p*-values for fixed effects were computed using Satterthwaite's approximation. Confidence intervals were calculated using the Wald method. SMD represents the fixed-effect estimate divided by the baseline SD of the outcome. Model equation: Mean Value ~ Session × Gender + (1 | ID). Gender was coded with female as the reference category.

**Table 3**
*Extended Linear Mixed-Effects Model Including Prior Experience*

| Concept |  |  |  | Fixed Effects | | | |  | | |  |
| --- | --- | --- | --- | --- | --- | --- | --- | --- | --- | --- | --- |
|  |  | Term | | | Estimate (β) | SE | 95% CI | | t | p | SMD |
| Stigma direct |  | Session | | | -0.05 | 0.06 | [-0.17, 0.07] | | -0.86 | .393 | -0.08 |
|  |  | Prior Experience | | | -0.18 | 0.07 | [-0.33, -0.04] | | -2.47 | .015 | -0.28 |
|  |  | Session x Prior Experience | | | 0.01 | 0.06 | [-0.11, 0.13] | | 0.12 | .905 | 0.01 |
| Stigma indirect |  | Session | | | -0.17 | 0.13 | [-0.43, 0.08] | | -1.35 | .181 | -0.12 |
|  |  | Prior Experience | | | -0.21 | 0.16 | [-0.53, 0.12] | | -1.25 | .213 | -0.14 |
|  |  | Session x Prior Experience | | | -0.07 | 0.13 | [-0.33, 0.18] | | -0.54 | .589 | -0.05 |
| Psychological services literacy |  | Session | | | 0.59 | 0.07 | [0.45, 0.72] | | 8.58 | < .001 | 0.80 |
|  |  | Prior Experience | | | 0.03 | 0.08 | [-0.12, 0.18] | | 0.38 | .705 | 0.04 |
|  |  | Session x Prior Experience | | | 0.03 | 0.07 | [-0.11, 0.16] | | 0.38 | .703 | 0.04 |
| General help-seeking intention |  | Session | | | 0.52 | 0.15 | [0.22, 0.82] | | 3.38 | .001 | 0.29 |
|  |  | Prior Experience | | | -0.13 | 0.18 | [-0.48, 0.23] | | -0.69 | .492 | -0.07 |
|  |  | Session x Prior Experience | | | 0.14 | 0.15 | [-0.17, 0.44] | | 0.88 | .382 | 0.07 |
| Source-specific help-seeking intention |  | Session | | | 0.04 | 0.01 | [0.02, 0.07] | | 3.86 | < .001 | 0.30 |
|  |  | Prior Experience | | | 0.00 | 0.02 | [-0.04, 0.03] | | -0.04 | .966 | -0.01 |
|  |  | Session x Prior Experience | | | -0.01 | 0.01 | [-0.03, 0.01] | | -0.71 | .481 | -0.06 |

*Note.* *p*-values for fixed effects were computed using Satterthwaite's approximation. Confidence intervals were calculated using the Wald method. SMD represents the fixed-effect estimate divided by the baseline SD of the outcome. Model equation: Mean Value ~ Session × Prior Experience + (1 | ID). Prior Experience was mean-centered.

**Table 4**
*Extended Linear Mixed-Effects Model Including Prior Knowledge*

| Concept |  |  |  | Fixed Effects | | | |  | | |  |
| --- | --- | --- | --- | --- | --- | --- | --- | --- | --- | --- | --- |
|  |  | Term | | | Estimate (β) | SE | 95% CI | | t | p | SMD |
| Stigma direct |  | Session | | | -0.05 | 0.06 | [-0.17, 0.07] | | -0.86 | .393 | -0.08 |
|  |  | Prior Knowledge | | | -0.14 | 0.09 | [-0.31, 0.04] | | -1.52 | .131 | -0.21 |
|  |  | Session x Prior Knowledge | | | 0.01 | 0.07 | [-0.13, 0.15] | | 0.16 | .873 | 0.02 |
| Stigma indirect |  | Session | | | -0.17 | 0.13 | [-0.43, 0.08] | | -1.35 | .182 | -0.12 |
|  |  | Prior Knowledge | | | -0.38 | 0.19 | [-0.75, -0.01] | | -2.00 | .048 | -0.26 |
|  |  | Session x Prior Knowledge | | | -0.04 | 0.15 | [-0.34, 0.26] | | -0.26 | .793 | -0.03 |
| Psychological services literacy |  | Session | | | 0.59 | 0.07 | [0.46, 0.72] | | 8.60 | < .001 | 0.80 |
|  |  | Prior Knowledge | | | -0.02 | 0.09 | [-0.20, 0.15] | | -0.27 | .788 | -0.03 |
|  |  | Session x Prior Knowledge | | | 0.06 | 0.08 | [-0.10, 0.22] | | 0.78 | .439 | 0.08 |
| General help-seeking intention |  | Session | | | 0.52 | 0.15 | [0.22, 0.82] | | 3.38 | .001 | 0.29 |
|  |  | Prior Knowledge | | | -0.19 | 0.21 | [-0.60, 0.23] | | -0.89 | .376 | -0.10 |
|  |  | Session x Prior Knowledge | | | 0.15 | 0.18 | [-0.21, 0.50] | | 0.81 | .422 | 0.08 |
| Source-specific help-seeking intention |  | Session | | | 0.04 | 0.01 | [0.02, 0.07] | | 3.86 | < .001 | 0.30 |
|  |  | Prior Knowledge | | | 0.00 | 0.02 | [-0.04, 0.04] | | 0.00 | .997 | 0.00 |
|  |  | Session x Prior Knowledge | | | -0.01 | 0.01 | [-0.03, 0.02] | | -0.44 | .658 | -0.04 |

*Note.* *p*-values for fixed effects were computed using Satterthwaite's approximation. Confidence intervals were calculated using the Wald method. SMD represents the fixed-effect estimate divided by the baseline SD of the outcome. Model equation: Mean Value ~ Session × Prior Knowledge + (1 | ID). Prior Knowledge was mean-centered.

**Figure 1**
*Residuals vs. Fitted Values Across Concepts*


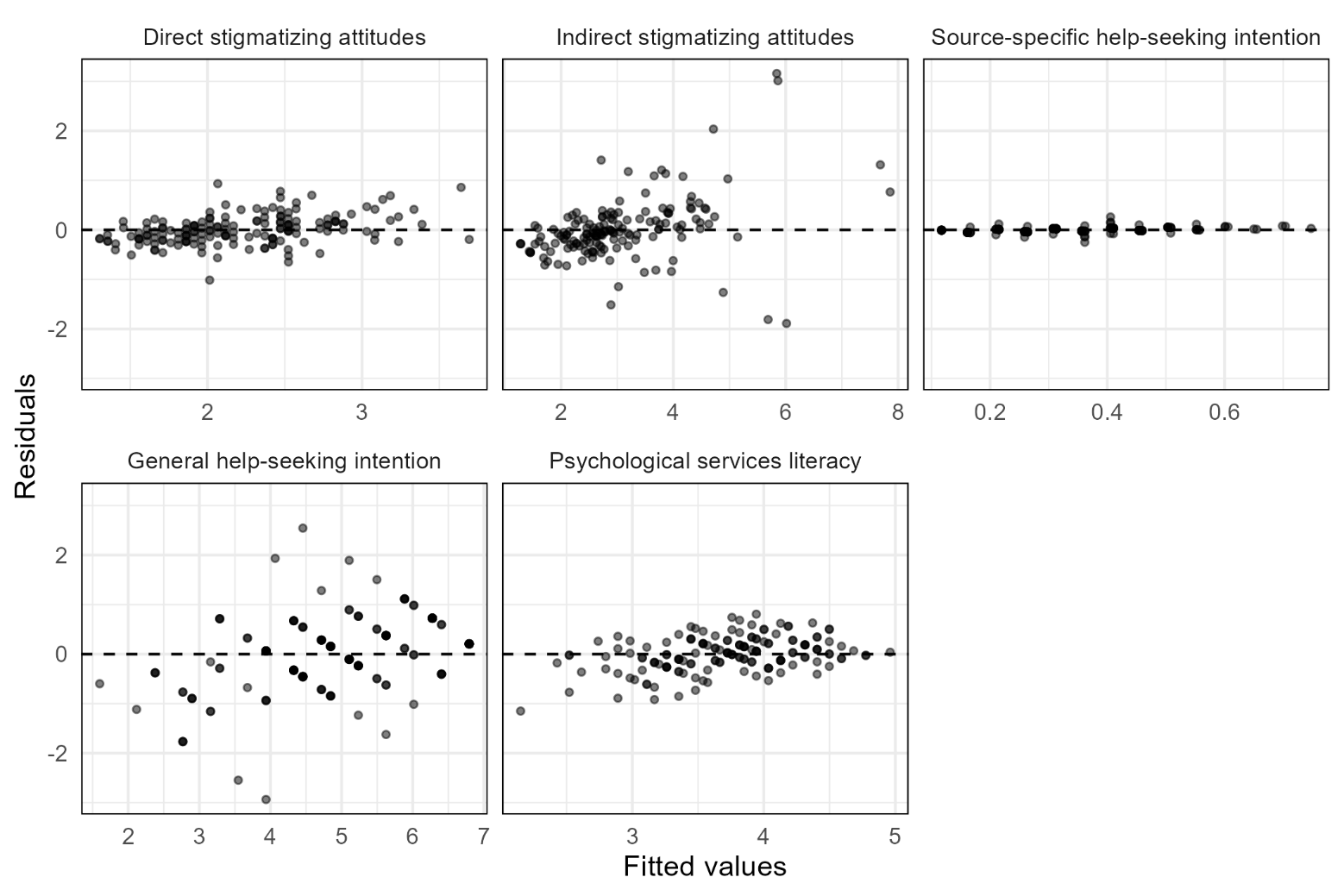


*Note.* Each panel represents one concept; scales differ across panels.

**Figure 2**
*Q-Q Plots of Model Residuals Across Concepts*


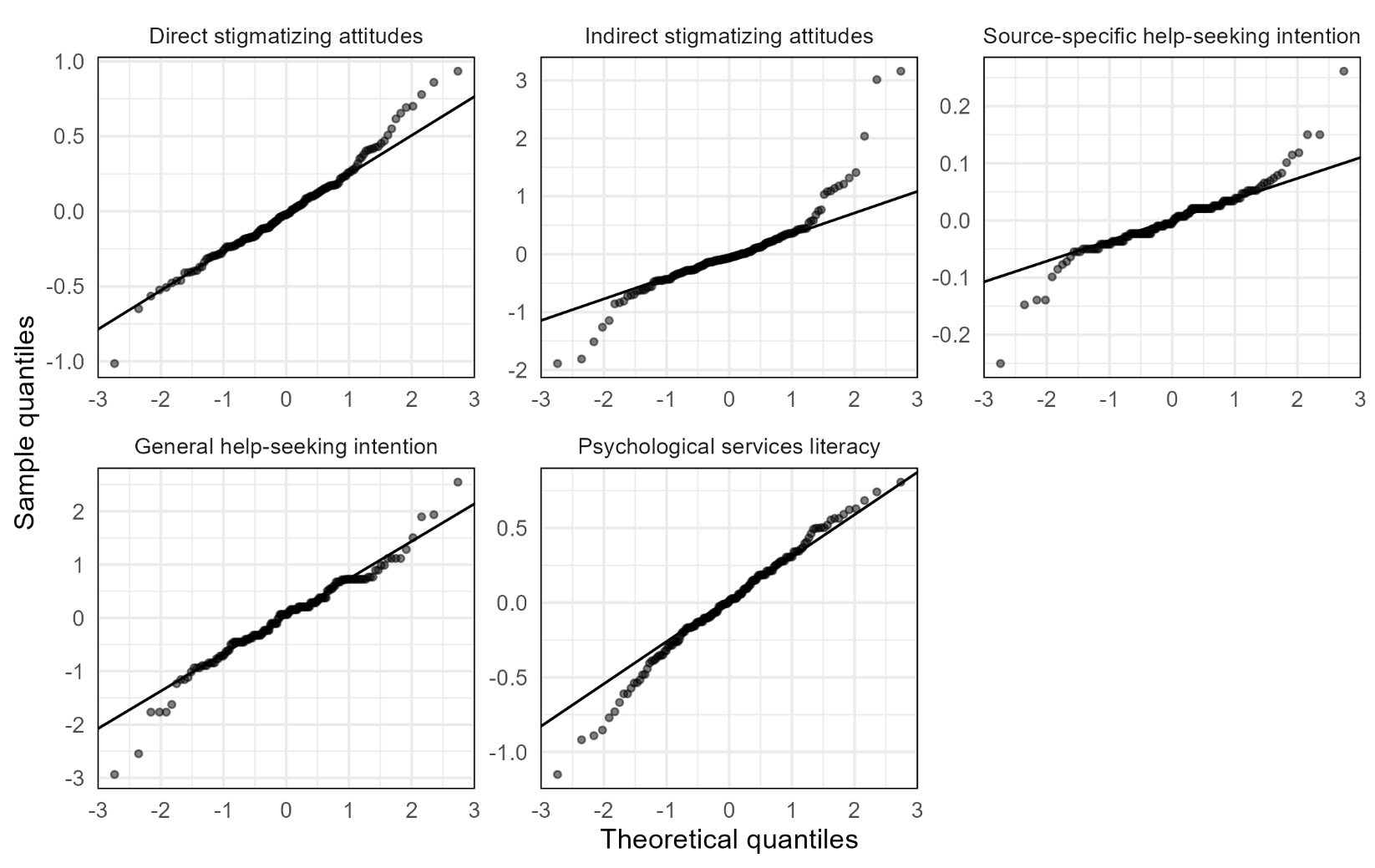


*Note.* Each panel represents one concept; scales differ across panels.

**Figure 3**
*Q-Q Plots of Random Intercepts Across Concepts*


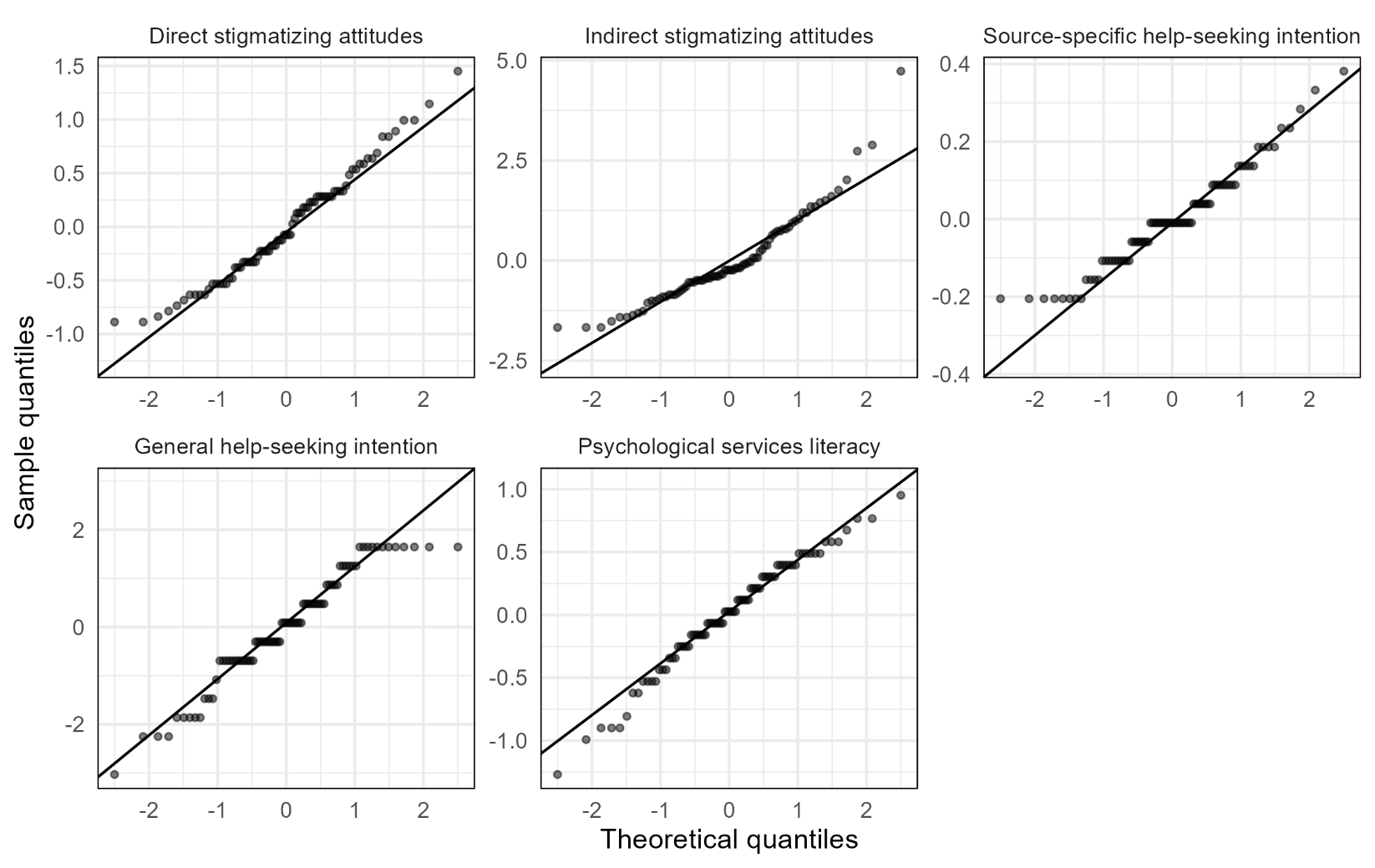


*Note.* Each panel represents one concept; scales differ across panels.
